# Phylogeography Study of the Siberian Apricot (*Prunus sibirica* L.) in Northern China Assessed by Chloroplast Microsatellite and DNA Makers

**DOI:** 10.3389/fpls.2017.01989

**Published:** 2017-11-21

**Authors:** Zhe Wang, Yanfei Zeng, Zhendong Zhang, Songbai Sheng, Ju Tian, Rongling Wu, Xiaoming Pang

**Affiliations:** ^1^Beijing Advanced Innovation Center for Tree Breeding by Molecular Design, National Engineering Laboratory for Tree Breeding, Key Laboratory of Genetics and Breeding in Forest Trees and Ornamental Plants, Ministry of Education, College of Biological Sciences and Biotechnology, Beijing Forestry University, Beijing, China; ^2^Center for Computational Biology, College of Biological Sciences and Biotechnology, Beijing Forestry University, Beijing, China; ^3^State Key Laboratory of Tree Genetics and Breeding, Chinese Academy of Forestry, Beijing, China; ^4^Inner Mongolia Hesheng Ecological Science and Technology Research Institute, Huhhot, China

**Keywords:** cpDNA, cpSSR, glacial refugia, Northern China, phylogeography, *Prunus sibirica*

## Abstract

There is evidence that a band of dry climate separated plants in East Asia into distinct northern and southern groups. However, few studies have focused on the arid belt in this region, especially with regard to plants. We analyzed genetic variation in 22 populations of Siberian apricot (*Prunus sibirica* L.), a temperate deciduous species distributed in this arid belt, using two chloroplast DNA (cpDNA) sequences, seven chloroplast microsatellite loci (cpSSRs), and 31 nuclear microsatellite loci (nSSRs), to study its phylogeography. Chloroplast data showed the complete fixation of two different genetic groups: the eastern and western groups. Genetic differentiation between the two groups was significant (F_ST_ = 0.90925, *p* < 0.01). This pronounced phylogeographic break was also indicated by nSSR data, but there were disparities regarding individual populations. An asymmetric gene flow via pollen and seeds likely resulted in discordance between the present-day geography of nuclear and chloroplast lineages. There was a distinct boundary between the two large groups, which were fixed for two of the most ancestral chlorotypes. Two populations with the highest chloroplast genetic diversity were located in the Yanshan Mountains and Jinzhou, considered to be the glacial refugia. The split of chloroplasts between the eastern and western groups was estimated to have occurred ~0.1795 Ma, whereas nuclear divergence occurred approximately 13,260 years ago. Linear regression analysis showed that climatic factors (annual precipitation and annual mean temperature) had a significant correlation with mean ancestry value (*P* < 0.05) indicated that they were potential factors for the formation of the two groups. In addition, this boundary was a contact zone between two groups from different refugia.

## Introduction

Phylogeographic studies have been used to investigate the effects of past climatic changes on the genetic structure of plant species, and allow inferences to be made about species evolution. The distribution ranges of plants and the genetic diversity within and among present-day populations of such organisms in the Northern Hemisphere have been affected deeply by climatic changes over the last 2 million years (Hewitt, 2000). It has long been thought that East Asia was an important mass refugium during climatic fluctuations over recent millions of years (Liu, 1988). Indeed, East Asia has recently been suggested to have been divided into distinct northern (northeast China, Japan, and Korea) and southern (southeastern and southern China) regions (Manos and Stanford, 2001; Milne and Abbott, 2002; Milne, 2006).

Instead of a physical barrier (mountains, ocean), an east-west arid belt which was between 35 and 45°N, has been thought to act as a climate barrier between the two regions (Tiffney and Manchester, 2001). This climate barrier has been subject to dynamic changes, which decreased and increased over geological time (Tiffney and Manchester, 2001; Guo et al., 2008). Bai et al. (2016) reported that the climate barrier in northern China persists today and acts as a divide. However, few studies have focused on the arid belt, and especially the plants in this region. The climate barrier was actually located at the intersection of the two regions, and evolution of species in this area, especially in northern China, would be expected to be more complex than that in the two regions.

Northern China is an appropriate area for assessing the effects of climate change on plant evolution. It is a botanically diverse region which is rich in both total species numbers and proportions of endemic species and underwent major climatic and geological changes during the last glacial maximum (LGM). Northern China (including north and northeast China) is covered with diverse plant biomes that range from tropical to cold forests and taiga (Gao et al., 2002). This region was considered to be an important part of the south-north vegetation transect in China, and palaeovegetation research has shown that it was subject to past climatic oscillations (Yu et al., 2000). Thus, northern China may serve as a model region for studying the migration of tree species during the LGM.

In Europe and North America, the locations of glacial refugia and postglacial migration routes of many plants and animals have been determined from a combination of fossil and genetic evidence (Huntley and Birks, 1983; Webb and Bartlein, 1992; Taberlet et al., 1998; Abbott et al., 2000). Qian and Ricklefs (2000) suggested that multiple refugia for forests might have existed and allowed species to persist across northern China during the LGM. Recently, there is increasing molecular evidence that supports this hypothesis. The phylogeographical patterns of conifer species (Chen et al., 2008) and temperate deciduous species (Tian et al., 2009) were examined and all of them were divided into different lineages, showing that multiple refugia were maintained in northern China. However, only a single glacial refugium, thought to have been located in the Changbai Mountains, is generally acknowledged (reviewed in Qiu et al., 2011). In addition to “traditional” refugia, small “cryptic” refugia or “microrefugia” might also have been widespread in northern China (Zeng et al., 2015). Bai et al. (2016) used Asian butternuts (*Juglans* section Cardiocaryon), covering the whole range of East Asia, to study the role of the climate barrier in diversification. Both nuclear and chloroplast data showed that the Yanshan Mountains contained a dividing line, separating the northeast and southwest lineages. Similar results have been described in other species, such as, walnut (*Juglans mandshurica*, Bai et al., 2010), Chinese oaks (*Quercus mongolica*, and *Quercus liaotungensis*, Zeng et al., 2011), and mono maple (*Acer mono*, Liu et al., 2014). However, the species studied are distributed primarily across the whole of East Asia.

Natural forests in the arid belt are dominated by broadleaf deciduous trees; the Siberian apricot (*Prunus sibirica* L.) is one of them. This temperate, deciduous, and wind-pollinated species is distributed widely across the mountainous areas of north and northeast China. It can also be found in eastern Siberia and Mongolia (Maynard, 1999). Siberian apricot trees are shrubs or high arbor. The height of the tree will be up to 5 m in the eastern coastal areas where the rain is plenty, but in in the west arid region it is only a few tens of centimeter. The seed kernels can be processed for biodiesel (Wang, 2012) and protein drinks (Sze-Tao and Sathe, 2000) and were also used to treat asthma, coughs, and infant virus pneumonia in traditional Chinese medicine. Moreover, Siberian apricot trees have often been used for afforestation in north China (Zhang et al., 2006). Although the Siberian apricot seeds are quite profitable, the commercial cultivar has been lacking and most of Siberian apricot seeds were collected from the wild Siberian apricot. Wild species play crucial roles in breeding programs because of their wide variability in terms of phonological, morphological, abiotic, and quality traits (Laidò et al., 2013). Siberian apricot is able to thrive under many types of harsh environmental conditions such as, low temperature, strong wind, low rainfall, and poor soil. During long-term evolution, the wild Siberian apricot populations generated a large number of variations. Vavilov (1992) suggested that the apricot had originated from three important centers: the Chinese, the Central Asian, and the Asia Minor centers. The Chinese center of origin may be the actual one for the Siberian apricot (Maynard, 1999). Using dominant inter-simple sequence repeat (ISSR), sequence-related amplified polymorphism (SRAP), and nuclear simple sequence repeat (nSSR) markers, Li et al. (2013) reported a relatively high level of genetic diversity, a low level of inter-population genetic differentiation, and a high level of intra-population genetic differentiation in this species. Moreover, a STRUCTURE analysis indicated that all Siberian apricot populations could be divided into two main groups. However, in our previous study, we clustered Siberian apricot populations into four clusters (Wang et al., 2014). There was a clear boundary between eastern and western clusters. Due to the limitations of the molecular marker system used, such information may be incomplete. Consequently, additional studies based on other marker systems and re-clustering may help to better reveal the “real” pattern of the population structure in this species. This also requires a more detailed study of individual clades in a robust phylogenetic framework and a more direct estimation of divergence times with accurate age estimation methods.

Generally, seed dispersal distance is much less than that of pollen, and population divergence due to genetic drift will be more marked for chloroplast DNA (cpDNA) than for nuclear DNA (Ennos, 1994). Indeed, cpDNA is considered to evolve very slowly, with low recombination and mutation rates (Wolfe et al., 1987; Clegg and Zurawski, 1992). Organelle markers could provide powerful tools for studying the phylogeography and migratory footprints of species (Avise, 2000).

In this study, we re-ran the STRUCTURE analysis using nSSR data we obtained, and used a set of chloroplast simple sequence repeats (cpSSRs) and cpDNA sequences to analyze Siberian apricot populations in China. The major aims of this study were to address the following questions: (1) How did nuclear DNA and cpDNA lineages of Siberian apricot distribute? (2) Does the Siberian apricot show any concordance between the geographical distribution of cpDNA and nuclear DNA lineages? (3) Did refugia exist in the Siberian apricot distribution regions? (4) What is the potential factor(s) of the formation of different lineages?

## Materials and methods

### Samples

**Figure 1:**
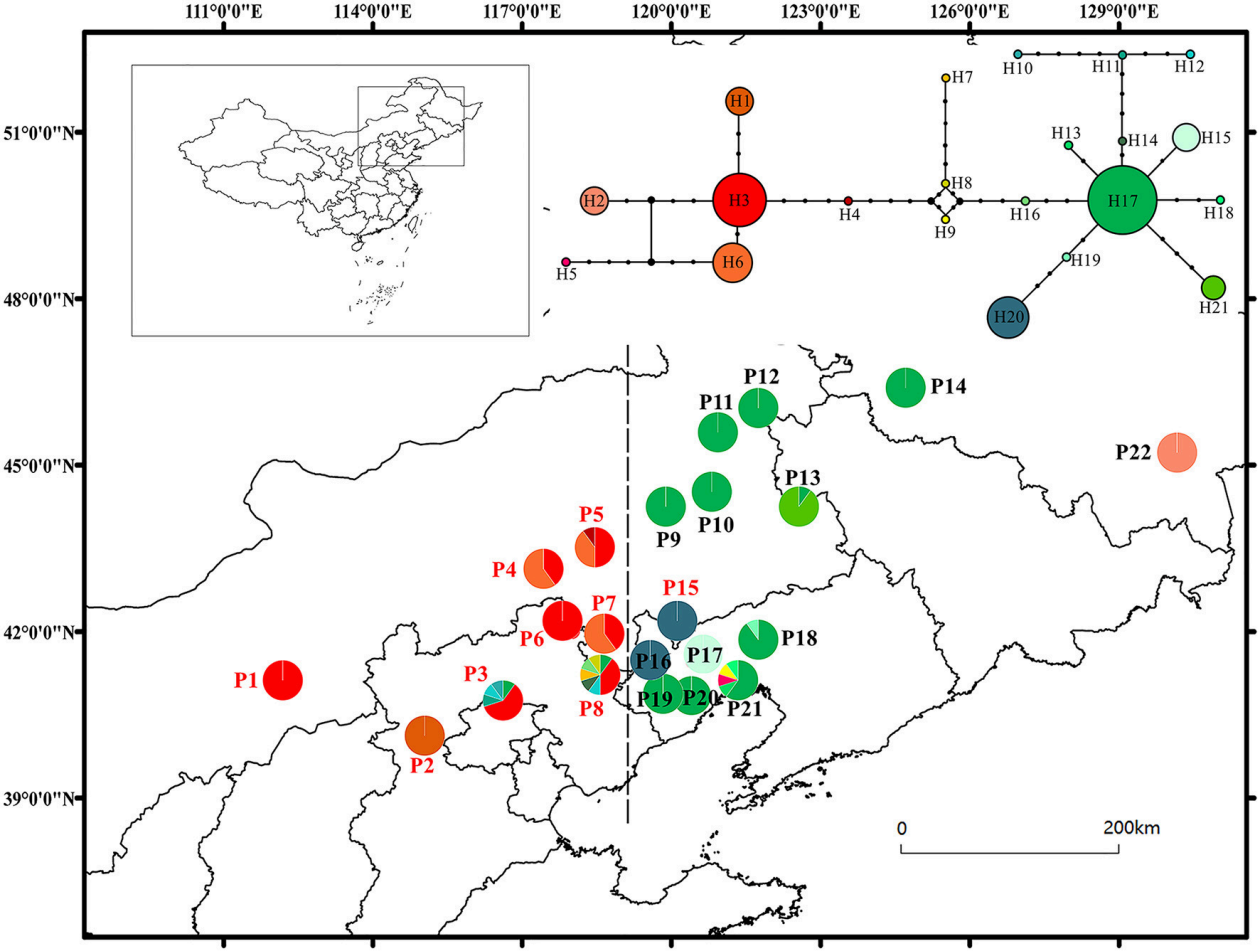
Map showing the locations of sampled populations, median-joining haplotype network and geographic distributions of the 21 chlorotypes found in the Siberian apricot. Population labels with different colors correspond to two genetic clusters identified by the program STRUCTURE: red, northern group; black, northeastern group. The dotted line is the boundary between the eastern and western groups. Circle sizes are proportional to the number of samples per chlorotype.

In total, 672 Siberian apricot individuals were collected from 22 populations covering the entire range of the natural distribution in China (Wang et al., 2014). For convenience of description, the numbers of populations were changed (Figure 1; Supplementary Table 1). In total, 222 individuals (10–11 individuals in each population) were selected for chloroplast marker analysis. Among the populations, three were designated as “semi-wild-type” because the trees were artificially grown from seeds collected randomly from the immediate area or near the region. The conditions and environment of growth of semi-wild populations were the same as wild populations. The phenotypes of the trees from semi-wild populations were very rich, and the results of molecular experiments also showed that the genetic diversity of the semi-wild populations was as high as that of the wild populations.

### DNA extraction

Young leaves were collected and placed immediately in Ziploc bags preloaded with colored silica gel to dry and preserve them for DNA extraction. Total genomic DNA was extracted from dry leaves using a modified version of the cetyl trimethylammonium bromide method (Doyle, 1987). The quality and concentration of the extracted DNA was determined by 1% agarose gel electrophoresis and ultraviolet spectrophotometry.

### cpSSR amplification and genotyping

We selected 19 cpSSR markers after an initial screening from 40 candidate cpSSR markers (Bryan et al., 1999; Weising and Gardner, 1999; Ohta et al., 2005; Cheng et al., 2006). Several representative amplicons of each allele were sequenced. The variation of some loci was not only due to the change in the number of repeats, but also related to indel in the flanking regions. Finally, seven loci with variation just in motif number were chosen to assay 222 individuals of Siberian apricot. They are TPScp1, TPScp3, TPScp4, TPScp11 (Ohta et al., 2005), ccmp3 (Weising and Gardner, 1999), ARCP5 (Cheng et al., 2006), and NTCP8 (Bryan et al., 1999). The forward primer of each pair was tagged with a section of the universal M13 sequence (5′-TGTAAAACGACGGCCAGT-3′) during synthesis. Amplification was performed in a 10-μL reaction mixture containing 1 μL of DNA template (10 ng/μL), 5 μL of 2 × Taq mix, 0.4 μL of the forward primer (1 μM), 1.6 μL of the reverse primer, 1.6 μL of M13 primer (1 μM) with a fluorescent label (FAM, HEX, ROX, or TAMRA), and 1.4 μL of double distilled H_2_O. The reaction conditions were: 94°C for 5 min, followed by 30 cycles of 94°C for 30 s, Ta (annealing temperature) for 1 min, and 72°C for 1 min, followed by 8 cycles of 94°C for 30 s, 53°C for 40 s, and 72°C for 45 s, with a final synthesis step at 72°C for 10 min. The products were separated with an ABI 3730XL DNA Analyzer using GeneScan-500LIZ as an internal marker (Applied Biosystems, Foster City, CA, USA). The fragments were sized using the Gene-Marker 1.75 software (SoftGenetics LLC, State College, PA, USA).

### cpDNA amplification and sequencing

Using universal primers, we conducted an initial screening for sequence variability of various chloroplast markers against 15 samples. The cpSSR haplotypes of the 15 samples were different from each other based on the result of completed cpSSR analysis. The intergenic spacers (IGSs) of the following pairs of genes were amplified using previously published primers: *atp*B-*rbc*L (Chiang et al., 1998; Zhou et al., 2010), *trn*L-*trn*F (Taberlet et al., 1991), *trn*Q-*rps*16 (Shaw et al., 2007), *acc*D-*psa*I, and *rpl*16F71-*rpl*16R15 (Small et al., 1998). The sequencing result showed that primers *atp*B-*rbc*L and *trn*Q-*rps*16 were available and sequences of the fragments were polymorphic. Then we used them for a large-scale survey of haplotype variation. PCR was performed in a 50-μL volume, containing 20–50 ng of plant DNA, 25 μL of 2 × Taq mix, and each primer (2 μM). Amplification was carried out under the following conditions: 94°C for 5 min, followed by 35 cycles of 94°C for 1 min, Tm for 1 min, a 2-min extension at 72°C, and a final synthesis step at 72°C for 10 min. PCR products were purified using a TIANquick Midi Purification Kit following the manufacturer's protocol (Tiangen). Purified PCR products were sequenced directly in both directions using the PCR primers on an ABI 3730XL DNA Analyzer (Applied Biosystems).

### Chloroplast data analysis

SSR variants at each locus were combined into haplotypes. cpSSR haplotype variation within populations was calculated with the following population diversity indices: the number of haplotypes (N), the effective number of haplotypes (Ne), the number of private haplotypes (Np), Nei's haplotype diversity (H), and the unbiased haplotype diversity (He) (Nei, 1987). Total genetic diversity H_T_, average within-population diversity H_S_, and two differentiation measures (G_ST_, N_ST_) were estimated for haplotypes and compared via a test with 1,000 permutations using Permut 2.0 software (Pons and Petit, 1996).

The *atp*B-*rbc*L and *trn*Q-*rps*16 sequences were combined into a complete sequence. Multiple alignments were performed with the ClustalX program (Thompson et al., 1997) and manually adjusted with BioEdit (ver. 7.0.4.1) software (Hall, 1999). Indels were generally placed so as to increase the number of matching nucleotides in a sequence position. Nucleotide diversity (Pi) (Nei, 1987) and haplotype diversity (Hd) (Nei and Tajima, 1983) were calculated using the DNA Sequence Polymorphism program (DnaSP) (Rozas et al., 2003). H_T_, H_S_, and two differentiation measures (G_ST_, N_ST_) were estimated with the same parameters as cpSSR analysis. All populations were grouped by performing spatial analysis of molecular variance (SAMOVA) using the SAMOVA software (ver. 1.0) (Dupanloup et al., 2002) that implements a simulated annealing approach to define groups of populations (K) that are geographically homogeneous and maximally differentiated from each other. The value of K was user-defined and set between 2 and 12, with 100 independent simulated annealing processes in each run. The maximum F_CT_-value, an indicator of genetic differentiation among population groups, was selected to identify the number of groups (K) for the “best” grouping of populations. Then, the observed genetic variation among and within the populations and groups was characterized by an analysis of molecular variance (AMOVA) using ARLEQUIN (ver. 3.5) software (Hamrick and Godt, 1996). Three hierarchical divisions were identified, based on the genetic variance: within populations, among populations within groups, and among groups using a non-parametric permutation procedure incorporating 10,000 iterations.

BEAST (ver. 1.8.2) software were implemented to estimate phylogenetic relationships and divergence times between cpDNA haplotypes (Drummond et al., 2012). The F81 nucleotide substitution model, selected with JModel Test (ver. 2.1.7) software (Darriba et al., 2012) was used. Given the lack of fossil records and substitution rates for the Siberian apricot, we used the average substitution rates of 4.62 × 10^−9^ substitutions per site per year (s s^−1^ y^−1^) reported for the angiosperm species 1–8.24 × 10^−9^ s s^−1^ y^−1^ to estimate the timescale of divergence (Richardson et al., 2001). We sampled all parameters once every 10,000 steps from 10^7^ Markov-coupled Markov chain (MCMC) steps. A uniform prior probability distribution was used to accommodate the uncertainty of the prior knowledge. We compared the outcomes of all five clock models with the Yule speciation process by using the TRACER program (ver. 1.6) (Rambaut and Drummond, 2007) with 1,000 bootstrap replicates. The strict clock model was better fit than the others which showed the lowest AICM value (Supplementary Table 2). Trees were then compiled into a maximum clade credibility tree using TREEANNOTATOR (ver. 1.8.2) (Drummond et al., 2012) and FIGTREE (ver. 1.4.2) (Rambaut, 2012) software to display mean node ages and highest posterior density (HPD) intervals at 95% (upper and lower) for each node and to estimate branch lengths and average divergence times. Historical demographic history of each clade was estimated with mismatch distributions analysis (MDA) (Rogers and Harpending, 1992) by using ARLEQUIN (ver. 3.5) software with 10, 000 permutaions. This analysis compared the expected frequencies of pairwise differences in haplotypes with those observed under a pure population growth model (Rogers and Harpending, 1992). A goodness of fit test was perform to assess the observed and expeted mismatch distribution and quantify the smoothness of the observed mismatch distribution based on the sum of squares deviations (SSD) and the raggedness index, respectively.

According to the known expected size and repeat motif of each cpSSR marker, the fragment size of all cpSSR data were transformed into sequences data. And both cpSSR and cpDNA are chloroplast markers, we spliced them into a mixed cpDNA sequence to complete the following analysis. H_T_, H_S_, G_ST_, and N_ST_ were estimated with the same parameters as above. To investigate relationships between haplotypes, we constructed a phylogenetic network tree for mixed cpDNA sequences using the median-joining model, implemented in Network (ver. 4.6.1.3) software (Bandelt et al., 1999). Site mutations and indels were assumed to evolve with equal probability, although they may exhibit different mutation rates when constructing a median-joining network. Each indel was considered to have originated independently. The SAMOVA and AMOVA analysis were performed with the same parameters as above.

### Nuclear SSR (nSSR) data analysis

In this study, we used the correlated allele frequencies model and the admixture model (Falush et al., 2003) to re-run the STRUCTURE program (ver. 2.3.3) Pritchard et al., 2000) based on the previous nSSR data with a more stringent parameter, which was 1,000,000 (in previous study was 100,000) Markov chain Monte Carlo repetitions after a burn-in period of 100,000 (in previous study was 25,000) iterations. The algorithm was run 20 (in previous study was 10) times for each K-value, from 1 to 11. The estimate of the best K was calculated as described by Evanno et al. (2005) using Structure Harvester (ver. 0.6.92) software (Earl and vonHoldt, 2012). The Clustering Markov Packager Across K (CLUMPAK, http://clumpak.tau.ac.il/index.html) program was used to simplify the comparison of clustering results from the STRUCTURE programs across all 20 repetitions of K and to determine the most likely number of clusters.

An approximate Bayesian computation (ABC) approach was used to infer the recent colonization history of the Siberian apricot. The STRUCTURE analysis revealed two clusters that corresponded to the western group (WG) and northeastern group (EG). To determine which among several scenarios of the history of divergence of populations from these regions was the most probable, we used the ABC procedure in DIYABC (ver. 2.0.4) software (Cornuet et al., 2014). We considered three demographic scenarios or models. In scenario 1, we assumed the ancestral effective population size varied at t2, and WG and EG diverged simultaneously at t1. In scenario 2, the ancestral effective population size varied at t2, and WG arose via divergence from the EG at t1. In scenario 3, the ancestral effective population size varied at t2, and EG arose via divergence from WG at t1 (Supplementary Figure 1). We used the MIGRATE software (ver. 3.6.11) (Beerli, 2006) to assess whether there was asymmetrical gene flow between the groups. The MIGRATE program (Beerli and Felsenstein, 1999) calculates maximum likelihood (ML) estimates for both effective population size and migration rates between pairs of populations using a coalescent approach. The program picked a random subset of individuals from the larger group, with the number of individuals in the subset being the same as in the smaller group. We relied on a maximum likelihood estimation and used three long chains (1,000,000), replicates = YES: 5, and randomtree = YES. To identify whether climatic factors potentially associated with the genetic structure and divergence between two groups, annual precipitation and annual mean temperature of each population were analyzed by regression analysis with the mean ancestry value of each populations, which were calculated by STRUCTRURE when *K* = 2 using R (R Core Team, 2016). The meteorological data were obtained from China Meteorological Data Center (http://data.cma.cn/).

## Results

### Chloroplast data

Seven perfect chloroplast microsatellite loci were used to assay 222 Siberian apricot individuals, resulting in 18 different alleles. In total, 15 unique cpSSR haplotypes were produced by combinations of the 18 different alleles. Details of the haplotype frequency and population genetic diversity are provided in Supplementary Tables 3, 4. Total genetic diversity H_T_, average within-population diversity H_S_ were 0.760 and 0.189 respectively. A permutation test showed that G_ST_ (0. 751) was significantly smaller than N_ST_ (0.860, *P* < 0.01).

The combined alignment of the two IGS (*atp*B-*rbc*L and *trn*Q-*rps*16) was 1,400 base pairs in length. Six nucleotide substitutions and five indels were found in the combined fragment which revealed eight cpDNA haplotypes (Table 1). Overall nucleotide diversity (Pi) (Nei, 1987) and haplotype diversity were 0.00120 ± 0.00005 and 0.57400 ± 0.00062, respectively. Total genetic diversity H_T_, average within-population diversity H_S_ were 0.652 and 0.090 respectively. N_ST_ (0.881) was not significant higher than G_ST_ (0.862). Five populations were polymorphic while the remaining 17 were fixed for a single haplotype (Supplementary Table 5). The analysis of spatial genetic structure for cpDNA variation using SAMOVA showed that the value of F_CT_ reached a plateau at *K* = 6 (Supplementary Figure 2). The grouping pattern of populations corresponding to *K* = 6 was: (P9, P10, P11, P12, P14, P15, P16, P18, P19, P20, P21); (P1, P2, P4, P5, P6, P7); (P3, P8); (P13); (P17); and (P22). Meanwhile, AMOVA of the six groups revealed that 91.70% of the variance was distributed among groups (Supplementary Table 6).

**Table 1:** Variable sites of mixed cpDNA sequences from 21 chlorotypes of Siberian apricot were identified.

**Mutation position**	***atp*****B–*****rbc*****L**					***trn*****Q-*****rps*****16**						**cpSSR**										
	**167**	**474**	**509**	**519**	**627**	**944**	**945**	**946**	**947**	**948**	**1221**	**1408**	**1410**	**1412**	**1419**	**1429**	**1438**	**1439**	**1448**	**1449**	**1457**	**1473**
H1	A	G	T	T	G	T	T	T	–	A	G	T	–	–	–	T	G	–	T	T	–	–
H2	A	G	T	T	G	T	T	T	–	T	G	–	–	–	–	T	–	–	–	–	–	–
H3	A	G	T	T	G	T	T	T	–	A	G	–	–	–	–	T	–	–	T	T	–	–
H4	A	G	T	T	G	T	–	–	–	A	A	–	–	–	–	T	–	–	T	T	–	–
H5	A	G	T	T	G	T	T	T	–	T	G	T	T	–	–	–	–	–	T	–	–	T
H6	A	G	T	T	G	T	T	T	–	A	G	–	–	–	–	T	–	–	T	T	–	T
H7	G	G	G	–	G	T	T	T	T	A	G	–	–	–	–	T	G	–	T	–	–	–
H8	G	G	G	–	G	T	–	–	–	A	A	–	–	–	–	T	–	–	T	–	–	–
H9	G	G	G	–	G	T	–	–	–	A	A	–	–	–	–	T	–	–	T	T	–	T
H10	G	G	G	–	G	T	T	T	T	A	G	T	T	–	–	–	G	G	T	T	–	T
H11	G	G	G	–	G	T	T	T	T	A	G	T	T	–	–	–	–	–	T	–	A	T
H12	G	G	G	–	G	T	T	T	T	A	G	T	T	T	–	–	–	–	–	-	A	T
H13	G	G	G	–	G	T	–	–	–	A	A	T	T	–	–	T	–	–	T	–	A	T
H14	G	G	G	–	G	T	T	–	–	A	A	T	T	–	–	–	–	–	T	–	A	T
H15	G	G	G	–	G	–	–	–	–	A	A	T	T	–	–	–	–	–	T	–	A	T
H16	G	G	G	–	G	T	–	–	–	A	A	–	–	–	–	–	–	–	T	–	A	T
H17	G	G	G	–	G	T	–	–	–	A	A	T	T	–	–	–	–	–	T	–	A	T
H18	G	G	G	–	G	T	–	–	–	A	A	T	T	T	–	–	–	–	–	–	A	T
H19	G	G	G	–	G	T	–	–	–	A	A	T	T	–	–	–	G	–	T	–	A	T
H20	G	G	G	–	G	T	–	–	–	A	A	T	T	T	–	–	G	–	T	–	A	–
H21	G	T	G	–	T	T	–	–	–	A	G	T	T	–	A	–	–	–	T	—	A	T

*cpDNA sequences are numbered from the 5′- to the 3′-end in each region*.

**Figure 2:**
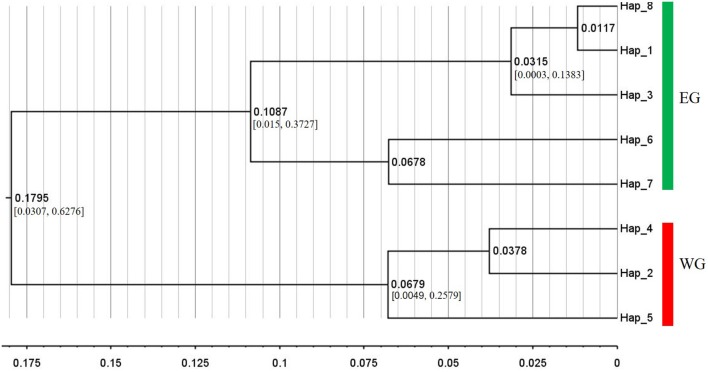
BEAST-derived chronograms of eight haplotypes of Siberian apricot based on two cpDNA fragments. Axis scale represents million years ago (Ma), and 95% highest posterior densities (HPDs) of nodes with posterior probabilities >0.9 are shown.

A phylogeny of cpDNA haplotypes showed lineage relationships with high statistical support (> 95%) were divided into two clades, one associated with the EG and the other with the WG (Figure 2, Supplementary Table 5). A point estimate for the divergence time between the two cpDNA clades dated to 0.1795 million years ago (Ma) (95% HPD: 0.0307–0.6276 Ma; Figure 2). Observed mismatch distribution of EG and WG were both multimodal (Supplementary Figure 3). Analysis of SSD and raggedness index tests suggested that the curves did not differ significantly from the expected mismatch distribution (Table 2). This indicated that the null hypothesis of recent demographic expansions was not rejected.

**Table 2:** Result of mismatch distribution analysis and neutrality test for two clades.

**Clade**	**SSD**	**P_SSD_**	**Raggedness index**	**P_Rag_**
WG	0.02435	0.24420	0.22849	0.54140
EG	0.01719	0.23110	0.40057	0.56650

In combination, the mixed cpDNA sequences identified 21 chlorotypes (Table 1). Sixteen of them (51 individuals) were private chlorotypes (Supplementary Table 1). Eight populations were polymorphic while the remaining 14 were fixed as a single chlorotype. The most abundant chlorotype was H17, which was found in 90 individuals (40.5%) from 12 populations that were located in Liaoning Hill, the Greater Khingan Mountains, the Northeast Plain, and the Yanshan Mountains. Chlorotype H3, discovered in seven populations, was the second most frequent and the only chlorotype in P6 and the most western population, P1. Population P2, P17, and P22 were fixed one private chlorotype: H1, H15, and H2, respectively. Thus, the average within-population diversity, H_S_ (0.196), based on chloroplast variation across all populations, was much smaller than the total genetic diversity, H_T_ (0.809). A permutation test showed that N_ST_ (0.871) was significantly higher than G_ST_ (0. 758, *P* < 0.01).

In total, 52 mutations and four median vectors were invoked to explain the network. Genetic relationships among the 21 chlorotypes, based on the network, are shown in Figure 1. The chlorotypes formed two haplogroups in the median-joining network. The right group included the most frequent chlorotype, H17, along with 11 less frequent chlorotypes whereas the left group comprised chlorotype H3 and five others. Each group exhibited a star-like topology with rare chlorotypes from the most widely distributed and commonly occurring chlorotypes.

The result of SAMOVA based on mixed cpDNA sequence showed that the value of F_CT_ reached a peak at *K* = 8. However, the value of F_CT_ reached a plateau when *K* = 3, and the range of the variance was weak after *K* = 3 (Supplementary Figure 4). It was reasonable that the populations clustered into three groups. The grouping pattern of populations corresponding to *K* = 3 was: (P9, P10, P11, P12, P13, P14, P17, P18, P19, P20, P21), (P1, P2, P3, P4, P5, P6, P7, P8, P22), and (P15, P16). SAMOVA of chloroplast data variation separated all populations into two large groups and one small one. The two large groups had a distinct boundary, which was the longitude lines of P16 (Figure 1). Moreover, AMOVA of the three groups revealed that 82.0% of the variance was distributed among the groups (Table 3).

**Table 3:** Analysis of molecular variance (AMOVA) of chlorotypes for populations and population groups of the Siberian apricot.

**Source of variation**	**d.f**.	**Sum of squares**	**Variance components**	**Percentage of variation**	**Fixation index**
Among groups	2	757.745	5.84610 Va	82.00	F_SC_: 0.49577
Among populations Within groups	19	134.310	0.63616 Vb	8.92	F_ST_: 0.90925
Within populations	200	129.400	0.64700 Vc	9.08	F_CT_: 0.82002
Total	221	1021.455	7.12926		

*The first analysis included three groups, grouped by SAMOVA through chloroplast data*.

*d.f., degree of freedom; F_ST_, variance among coefficient of individual relative to the total variance; F_SC_, variance among subpopulations within groups; F_CT_, variance among groups relative to the total variance*.

### nSSR data

**Figure 3:**
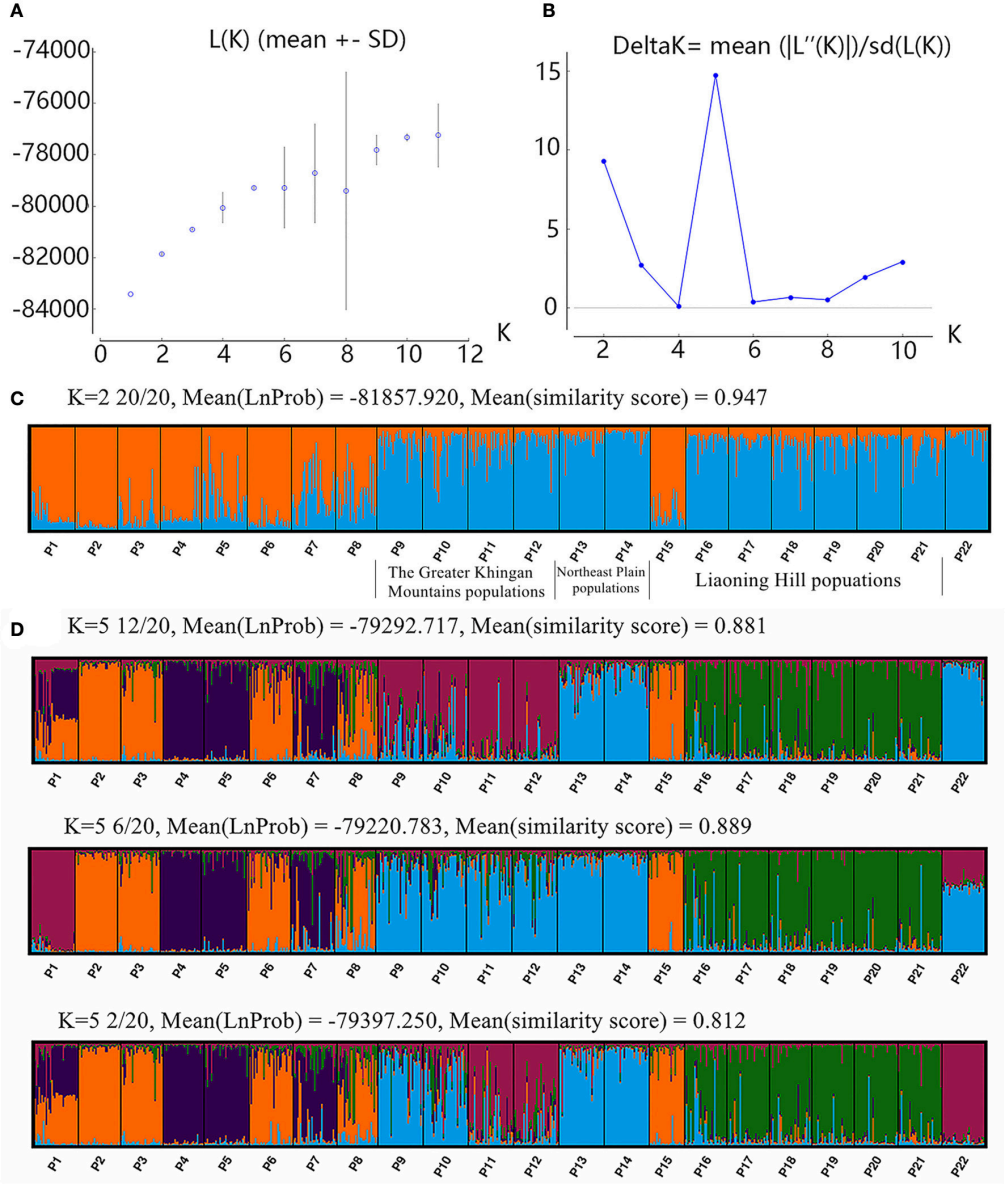
Bayesian inference of the number of clusters (K) of the Siberian apricot. K was estimated using **(A)** the posterior probability of the data, given each K (20 replicates). **(B)** The distribution of ΔK. **(C)** The CLUMPAK main pipeline of two clusters detected from STRUCTURE analysis. **(D)** The CLUMPAK main pipeline of five clusters which were detected from STRUCTURE analysis.

Results of the STRUCTURE analysis showed that the estimated logarithm of probability of ln P(K) showed an upward trend until *K* = 11. The data increased linearly from *K* = 1 to *K* = 5, and then showed certain amplitude fluctuations with large standard deviations except at *K* = 10 (Figure 3A). The values of ΔK were not very large. The highest ΔK occurred at *K* = 5 (Δ*K* = 14.72), and the second largest ΔK was at *K* = 2 (Δ*K* = 9.30). However, the absolute values of ΔK were very small (Figure 3B). The CLUMPAK main pipeline showed that all clustering results were unstable, with more than one clustering case for each K except *K* = 2 (Figure 3C, Supplementary Figure 5). There were three clustering cases when *K* = 5, although it had the highest ΔK (Figure 3D). The biggest differences among the three clustering cases were those of the Greater Khingan Mountains populations (P9, P10, P11, and P12), the Northeast Plain populations (P13 and P14), and the extreme populations (P1 and P22) clustered into different clusters. It seemed more likely that this cluster had the structure of a sub-cluster. Thus, it seemed more reasonable that all populations were clustered into two groups: the EG (P9, P10, P11, P12, P13, P14, P16, P17, P18, P19, P20, P21, P22) and the WG (P1, P2, P3, P4, P5, P6, P7, P8, P15) (Figure 1).

**Figure 4:**
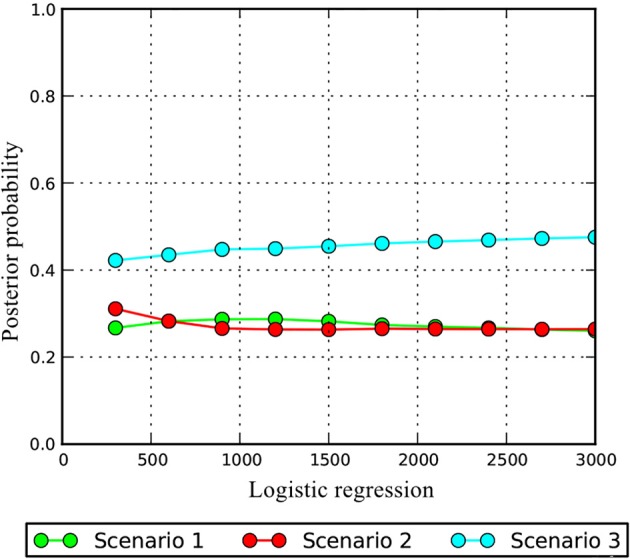
Posterior probability of the three scenarios.

**Figure 5:**
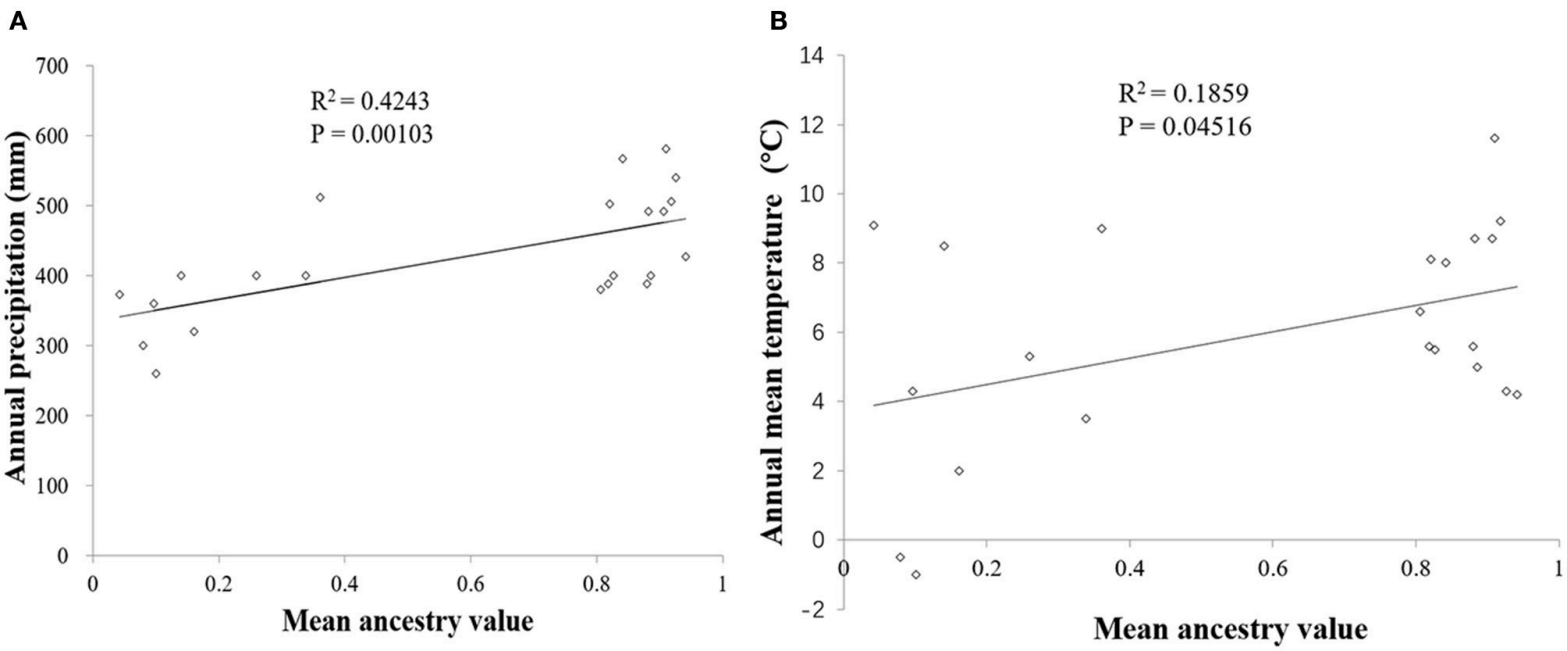
Regression analysis between climatic factors and the mean ancestry value of each populations. **(A)** Annual precipitation and the mean ancestry value. **(B)** Annual mean temperature and the mean ancestry value.

A comparison of posterior probabilities of the three scenarios using local linear regression indicated that scenario 3 was the most likely scenario, with a posterior probability of 0.48 (Figure 4). For scenario 3, the median values of t1 (the time the EG arose via divergence from the WG) and t2 (the time when the ancestral effective population size varied) were 663 generations and 4,410 generations, respectively (Supplementary Figure 6, Table 4). The median values of the effective population sizes of N1, N2, and Na were 11,500, 5,660, and 4,310, respectively. Estimates of gene flow, calculated with the MIGRATE software, and based on all 31 nSSRs data indicated high levels of gene flow between the two groups. The unidirectional estimate of 4 N*m*_EG → *WG*_ was 344.75, and that of 4 N*m*_WG → *EG*_ was 322.86. Linear regression analysis showed that the annual precipitation had a significant correlation with mean ancestry value (*P* = 0.00103 < 0.01) whereas annual mean temperature had a significant correlation with mean ancestry value at P = 0.05 level (Figure 5).

**Table 4:** Prior and posterior distributions for each parameter of scenario 3 obtained using DIYABC analysis.

**Parameter**	**Mean**	**Median**	**Mode**	**q025**	**q050**	**q250**	**q750**	**q950**	**q975**
N1	1.30E+04	1.15E+04	1.04E+04	3.24E+03	3.93E+03	7.47E+03	1.74E+04	2.63E+04	2.79E+04
N2	5.79E+03	5.66E+03	5.11E+03	2.12E+03	2.52E+03	4.20E+03	7.40E+03	9.23E+03	9.69E+03
t1	6.54E+02	6.63E+02	7.37E+02	2.47E+02	2.95E+02	4.92E+02	8.24E+02	9.62E+02	9.83E+02
t2	4.75E+03	4.41E+03	2.09E+03	6.60E+02	8.76E+02	2.29E+03	7.08E+03	9.31E+03	9.65E+03
Na	4.63E+03	4.31E+03	6.70E+02	3.34E+02	5.10E+02	2.18E+03	7.11E+03	9.51E+03	9.74E+03
Âμmic_1	5.19E−04	4.92E−04	3.81E−04	2.09E−04	2.43E−04	3.59E−04	6.60E−04	8.99E−04	9.45E−04
pmic_1	3.51E−01	3.05E−01	1.23E−01	1.10E−01	1.23E−01	1.93E−01	4.77E−01	7.22E−01	7.65E−01

## Discussion

### Chloroplast DNA diversity of the Siberian apricot

Our previous study showed a high level of genetic diversity in the Siberian apricot, based on nSSR data (Wang et al., 2014). The cpSSRs also showed a relatively high level of genetic diversity (H_T_ = 0.760 for cpSSR). The genetic diversity of cpDNA (H_T_ = 0.652) showed a relatively lower level of genetic diversity than those in other deciduous tree species, such as, *Prunus spinosa* (H_T_ = 0.824, Mohanty et al., 2000) and *Pyrus betulaefolia* (H_T_ = 0.826, Zong et al., 2014). Although a relatively low level of total diversity, based on cpDNA, was recorded in the Siberian apricot, it was enough to reflect the long evolutionary history and wide distribution of this species. Moreover, the result of mixed cpDNA sequences showed a much higher genetic diversity (H_T_ = 0.809 vs. 0.670) than that of 170 plant species (Petit et al., 2005). Because the Siberian apricot is distributed in large areas of China, across 18 longitudes and in a large range of altitudes (87–1,334 m), the habitats of this species are complex and diverse. Moreover, combined use of two kinds of markers can provide much more information about genetic diversity and population structure than that provided by each marker alone. The populations (P3, P8, and P21) showing the highest level of genetic diversity as revealed by mixed cpDNA sequences were inconsistent with those (P10, P16, and P20) revealed by nSSR data (Wang et al., 2014). This may be because the genetic diversity of nuclear DNA is determined mainly by gene flow and environmental factors. However, unlike nuclear DNA, the genetic diversity of cpDNA is determined by the length of evolutionary time and the composition of lineages. Moreover, gene flow of chloroplast maternally inherited genes occurs via seed dispersal and thus is more restricted than that of nuclear genes, which are inherited biparentally and dispersed by pollen and seeds (Birky et al., 1983; Ennos, 1994). Results of the chloroplast data analysis showed that P16, a semi-wild population, and P15 shared the same chlorotype, indicating that they had a common origin. P16 and P19 were from the same region and less than 17 km apart. Our previous study suggested that the seeds from P16 were local (Wang et al., 2014).

### Population structure of nuclear and chloroplast data

G_ST_ explains genetic variation over all populations whereas N_ST_ interprets the genetic differentiation influenced by haplotype frequency and the genetic distance between haplotypes. Here, the value of N_ST_ (0.871) was significantly higher than that of G_ST_ (0.758) using mixed cpDNA sequence data, indicating the presence of a phylogeographic structure. The grouping of populations produced by SAMOVA of mix-sequences variations strikingly similar to that of STRUCTURE, based on nSSRs (Figure 1). SAMOVA grouped all populations into two large groups and one small group that included P15 and P16. Additionally, the network based on mixed cpDNA sequence data showed that all chlorotypes from each group were closely related. The chlorotype H20, which was fixed in P15 and P16, was located in the right part of the network, indicating the EG group, and it had a close relationship with the most frequent chlorotype, H17 (Figure 1). This indicated that P16 and P15 still belonged to the EG. By comparison, the STRUCTURE analysis also clustered all populations into two groups. The difference was that P22 was clustered into the EG and P15 was clustered into the WG. The discordance between the present-day geography of nuclear DNA and cpDNA lineages was likely caused by high and asymmetric levels of gene flow via pollen and seeds. We suggest that this kind of asymmetrical gene flow has occurred in the two populations. Although P15 was located in the EG, it had more gene exchange via pollen with the WG than with the EG. Bai et al. (2010) reported that asymmetrical gene flow occurred between the two large groups, with the northern group (the WG in our study) nuclear genome being introduced into the northeastern group (the EG in our study) via pollen. The monsoon was likely the main factor for this asymmetrical gene flow. Our results also showed whole gene flow between the two groups was a little asymmetrical. P22 was far away from the other groups in geographic distance, but showed a close relationship in nuclear genetic distance (Wang et al., 2014). However, this geographic distance was too far away for spread via seeds.

The presence of a large number of private chlorotypes, and two star-like topologies exhibited in chlorotype network are usually indicative of historical expansion (Slatkin and Hudson, 1991). Such an expansion is also supported by the results of MDA which accepted the null hypothesis indicated EG and WG experienced recent demographic expansions in the past. According to coalescence theory, the most common chlorotypes, H3, and H17, which were dispersed over a large area and located at the center of the left and right part of chlorotype network, seemed to be the most ancestral chlorotypes (Posada and Crandall, 2001). Chlorotype H17 was the most common chlorotype in the EG and the only chlorotype in populations P9, P10, P11, P12, P14, P19, and P20, within which the absence of mixed cpDNA sequences variation indicated that they were probably derived from adjacent population(s) through colonization, rather than being relicts after genetic drift or a founder event. If the latter was true, the populations would probably be fixed for different chlorotypes rather than the same one (Zhang et al., 2005; Liu et al., 2013). The latter seemed to apply more to the formation of the WG, which was filled with the second most frequent chlorotype, H3, although populations P1 and P6 were completely fixed for it.

### Refugia and microrefugia for the Siberian apricot

Multiple geographically isolated refugia existed for forests across East Asia during the LGM and possibly the previous glacial maxima, which promoted intraspecific divergence, leading to speciation and high diversification of plants in this region (Qian and Ricklefs, 2000). The result of phylogenetic analysis for *Ostryopsis davidiana* suggested that multiple refugia were maintained across the entire distribution region of this species in northern China (between 31 and 44°N) during the LGM (Tian et al., 2009). Our survey of mixed cpDNA sequence variation throughout the geographical distribution area of the Siberian apricot resolved two large phylogeographic groups within this species of the deciduous forest. This finding suggested that, in the past, its distribution was fragmented into two isolated refugia. Consistent with the temperate-deciduous tree species *Juglans manshurica*, the survey of both cpDNA and nSSR variation showed two different lineages, suggesting that the species distribution was fragmented into two independent refugia, the Qingling/Taihang Mountains and the Changbai Mountains, in the past (Bai et al., 2010). Additionally, *P. betulaefolia*, a cool-temperate deciduous tree of the Rosaceae, survived the LGM or earlier cold periods in several isolated refugia in northern China (Zong et al., 2014). However, high levels of pollen flow between refugia in wind-pollinated trees might have eliminated the genetic imprints of Pleistocene refugial isolation (Liepelt et al., 2002). Populations in refugia usually display more genetic diversity and exclusive haplotypes than migratory populations (Hewitt, 2000, 2004). Fortunately, we found populations, P3, P8, and P21, with the highest chloroplast genetic diversity that seemed to be relicts of Quaternary glaciation and played important roles in shaping the genetic composition of adjacent populations. P3 and P8 were located in the Yanshan Mountains, which have been considered as refugia. The versatile biome in this area may have provided protective environments that preserved the genetic diversity of the Siberian apricot. We believe that the Taihang Mountains were also an important survival area during the cold period, although there is no natural Siberian apricot population distributed in the Taihang Mountains today. That was likely because the climate in this region did not reach the chilling requirement for the Siberian apricot in postglacial times. If a population comprises a mixture of haplotypes from larger phylogeographic groups, it may have resulted from the admixture of divergent lineages from separate refugia (Petit et al., 2003). Geographically, P8 was located at the fringe of two large groups, and this situation provides a high chance to gain seeds from the EG. Thus, we cannot exclude the possibility that P8 with a high level of cpDNA genetic diversity was the result of seeds derived from different, large groups. P21 was also detected within a WG lineage; this might be because WG seeds spread eastward through the Bohai sea. Nevertheless, this does not affect the suggestion that P21 was a long-term glacial survivor.

Based on the weak or absent genetic structure among the northeast China populations of temperate forest deciduous species, these phylogeographic studies suggest that the current distributions of cool-temperate species in northeast China (the EG in our study) originated from a single glacial refugium, which was located in the Changbai Mountains (Hu et al., 2008; Bai et al., 2010; Liu et al., 2014; Zong et al., 2014). Recently, it has also been proposed that many species, in fact, survived in smaller pockets at some distance from their core refugial areas, so-called microrefugia, from where they recolonized surrounding areas once climatic conditions improved (Stewart and Lister, 2001; Hampe and Jump, 2011). Some species might even have survived only in such small, scattered microrefugia. Bai et al. (2016) suggested that several microrefugia were possible between the Changbai and Xiaoxing'an mountains, ranging from 44 to 47°N. We should consider the most eastern population, P22, to the north of the Changbai Mountains, and fixed for chlorotype H2. The network analysis showed that H2 was in the left part of the network and had a close relationship with H3, which was most common in the WG (Figure 1). This indicated that chlorotype H3 once appeared in all of the distribution area of the Siberian apricot. As a refuge, P22 retained only chlorotype H2 during the LGM, but did not colonize other regions in postglacial times.

### The distinct boundary between the two large groups

The DIYABC analysis showed that during the time the EG arose via divergence from the WG, there were only 663 generations. Because the Siberian apricot begins to produce apricots at ~5 years and its longevity can extend beyond 40 years, we considered 20 years to represent a reasonable generation time, and it converted the divergence time t1 to 13,260 years ago, which was the end of the LGM. Because microsatellites have some inherent problems such as, uncertain mutation model and homoplasy (Selkoe and Toonen, 2006), this divergence time should be considered with caution. On a large time scale, homoplasy at microsatellite loci tended to underestimate divergence time (Takezaki and Nei, 1996). Indeed, the assumption of no gene flow between each branch in DIYABC analysis also probably underestimated the divergence time (Leaché et al., 2013). The split time (0.1795 Ma) of the two groups, based on cpDNA, which was much earlier than that based on nuclear data should be more reliable. The most likely scenario showed that the WG was more ancient than the EG. That is, chlorotype H3 and closely related chlorotypes are older than the most frequent chlorotype H17. Thus, P22 was the most ancient population in the EG. This further suggests that P22 was in a microrefugium during the LGM.

The distinct boundary contains a complex topography. The northern part of the boundary, between P5 and P9, which are both located in the Greater Khingan Mountains, was without any geographical barrier. The southern part was located in the Liaoxi corridor, where the site type is coastal hilly plains. Only the middle part coincided with the Nuluerhushan Mountains, which are the eastern extension of the Yanshan Mountains. Similar division was observed with temperate deciduous trees, such as, walnut (*J. mandshurica*; Bai et al., 2010), Chinese oak (*Q. mongolica* and *Q. liaotungensis*; Zeng et al., 2011), mono maple (*A. mono*; Liu et al., 2014), and the Asian butternut (*Juglans* section *Cardiocaryon*; Bai et al., 2016). These results indicate that the cryptic boundary was located in the Yanshan Mountains and neighboring areas. Moreover, Bai et al. (2016) calculated that the divergence time of the closely related Asian butternuts dated back to the Pliocene. This suggested that the distinct boundary appeared far earlier than the LGM. The results of BEAST analysis showed that the divergence time (0.1795 Ma) of EG and WG was also before the LGM. The divergence time was in Middle Pleistocene which covered a period of repeated glaciation (Head and Gibbard, 2005). The factors led the divergence of the two groups warrant a further study. For the current population distribution pattern, we suspect that climate was an important factor in the formation of this boundary. The WG is under a temperate continental climate, whereas the climate of the EG is temperate monsoon. Although the belts of these two climates have shown dynamic changes in different geological periods, the north-south boundary between them was always near the Yanshan Mountains (Guo et al., 2008). Rainfall and temperature, which are especially important for temperate deciduous tree growth, were very different between them. The significant correlations between the two climatic factors and mean ancestry values proved the inference in a certain extent. Furthermore, the climate would affect the soil formation that effect tree growth indirectly (Prescott, 1950). Thus, a distinct boundary was located in the Yanshan Mountains where trees from the northeast China refugia and north China refugia formed a recent secondary contact zone (Zeng et al., 2011). Indeed, this area was likely a secondary contact zone for northern and southern regions of the whole of East Asia.

## Conclusions

Although regional asymmetric levels of gene flow, via pollen, and seeds, resulted in slight discordance between the present-day geography of nuclear and chloroplast lineages for the Siberian apricot, our nuclear and cpDNA results support the existence of a distinct boundary between the eastern and western groups of northern China during the LGM. According to the linear correlation analysis, the potential factor for the formation of the two groups was likely the different climatic factors such as, rainfall and temperature. Each of the two groups had its own refugia during the LGM. Because of the extremely high levels of chloroplast genetic diversity, the Yanshan Mountains, and Jinzhou (P21) were likely the refugia for the WG and EG, respectively. Moreover, an isolated microrefugium (P22), near the Changbai Mountains, was suggested within the EG. Overall, apart from geographic barriers, our findings highlight the importance of climate in the present-day distribution of temperate deciduous trees in northern China.

## Accession numbers

All cpDNA sequences were deposited in GenBank under the accession numbers KY000823-KY000832.

## Author contributions

ZW, RW, and XP planned and designed the research. ZW, ZZ, JT, and SS performed experiments and analyzed data, and ZW, YZ, and XP wrote the manuscript.

### Conflict of interest statement

The authors declare that the research was conducted in the absence of any commercial or financial relationships that could be construed as a potential conflict of interest.

## Supplementary material

Supplementary Figure 1 The three scenarios tested in DIYABC analysis. In these scenarios, t represents the time-scale in terms of the number of generations, and N1 and N2 represent the effective population sizes of the northeastern and northern groups.

Supplementary Figure 2 F_CT_ distribution of combined cpDNA data.

Supplementary Figure 3 Mismatch distribution for two clades.

Supplementary Figure 4 F_CT_ distribution of mixed cpDNA sequence data.

Supplementary Figure 5 The CLUMPAK main pipeline of all clustering.

Supplementary Figure 6 Prior and posterior distributions for each parameter in scenario 3, obtained using DIYABC analysis.
