# Supplementary figures and images for: Phylogeography Study of the Siberian Apricot (Prunus sibirica L.) in Northern China Assessed by Chloroplast Microsatellite and DNA Makers
Source: Front Plant Sci. 2017 Nov 21;8:1989. doi: 10.3389/fpls.2017.01989 (PMC5702509; doi:10.3389/fpls.2017.01989)

Scenario 1

- N1
- N2
- Na
- N1+N2

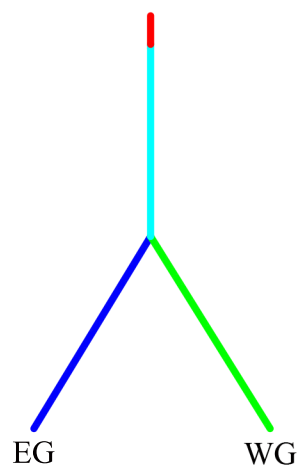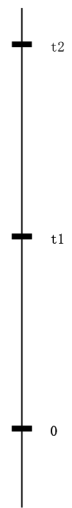

Scenario 2

- N1
- N2
- Na

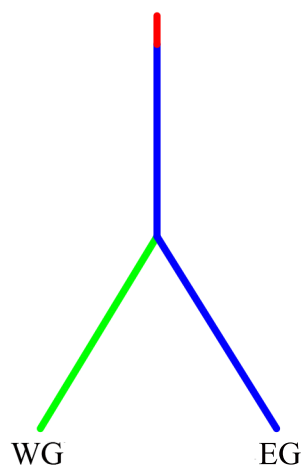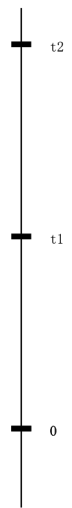

Scenario 3

- N1
- N2
- Na

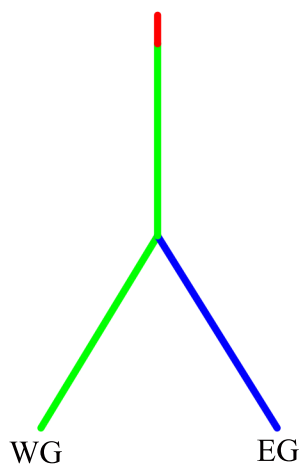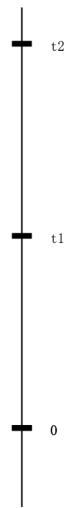

Supplement: Supplementary Figure 1 — The three scenarios tested in DIYABC analysis. In these scenarios, t represents the time-scale in terms of the number of generations, and N1 and N2 represent the effective population sizes of the northeastern and northern groups. [file Image1.PDF]

# SAMOVA

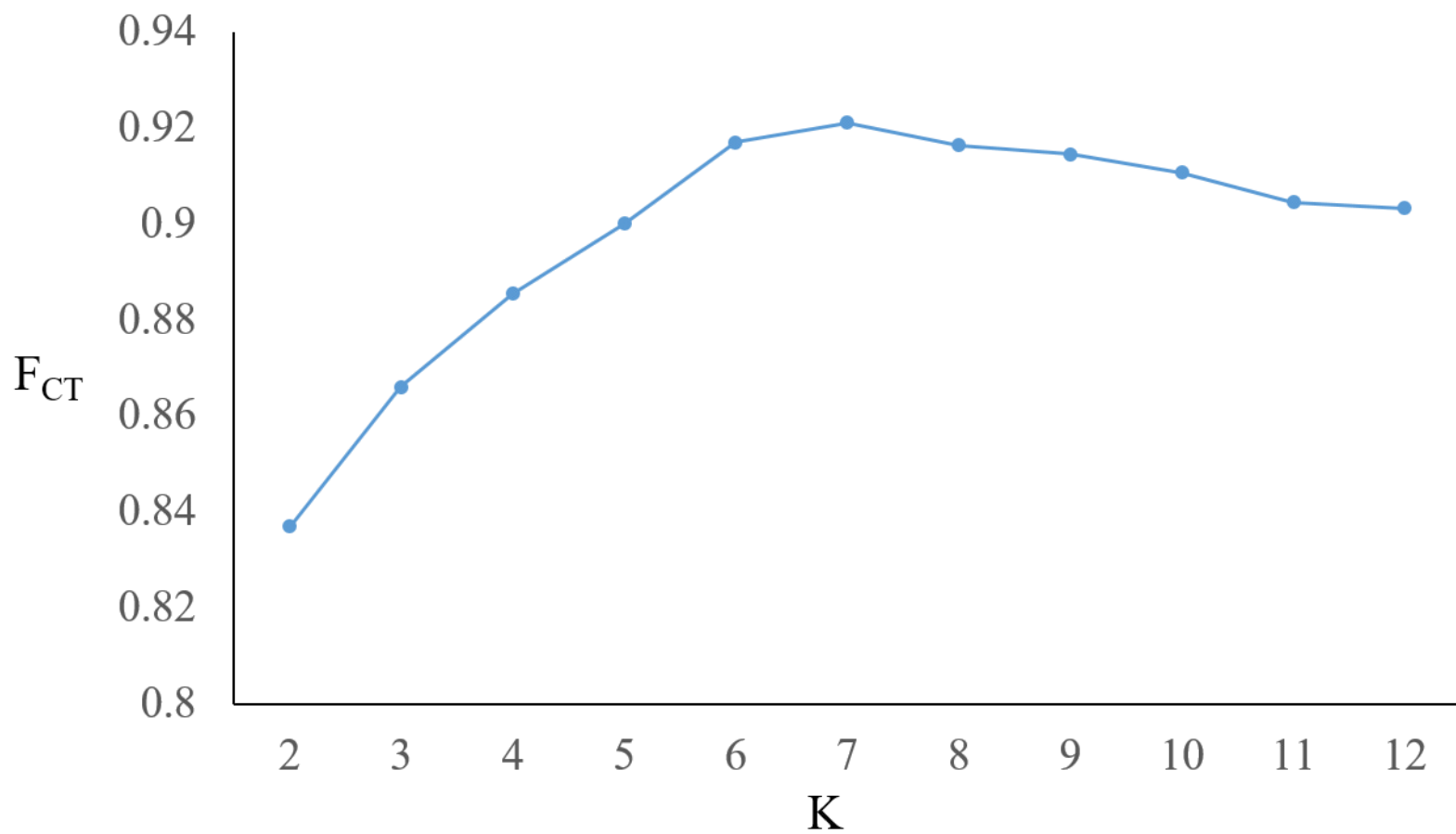

Supplement: Supplementary Figure 2 — FCT distribution of combined cpDNA data. [file Image2.PDF]

## EG

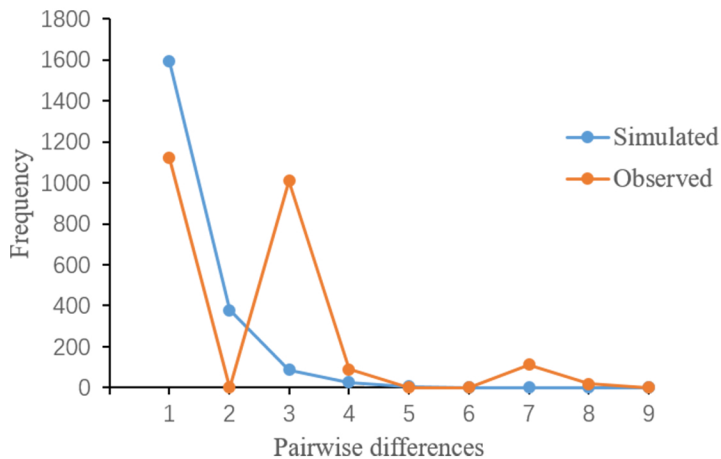

## WG

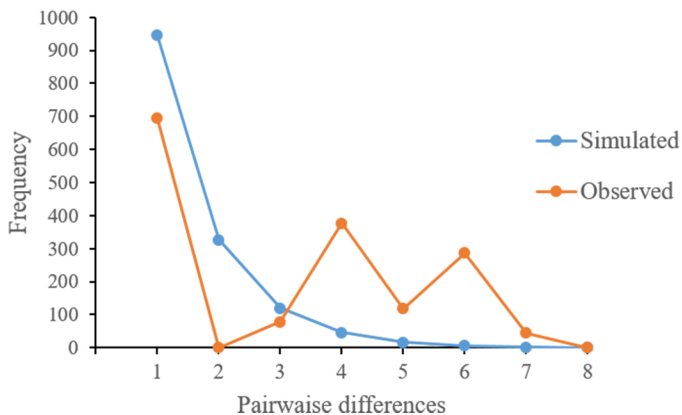

Supplement: Supplementary Figure 3 — Mismatch distribution for two clades. [file Image3.PDF]

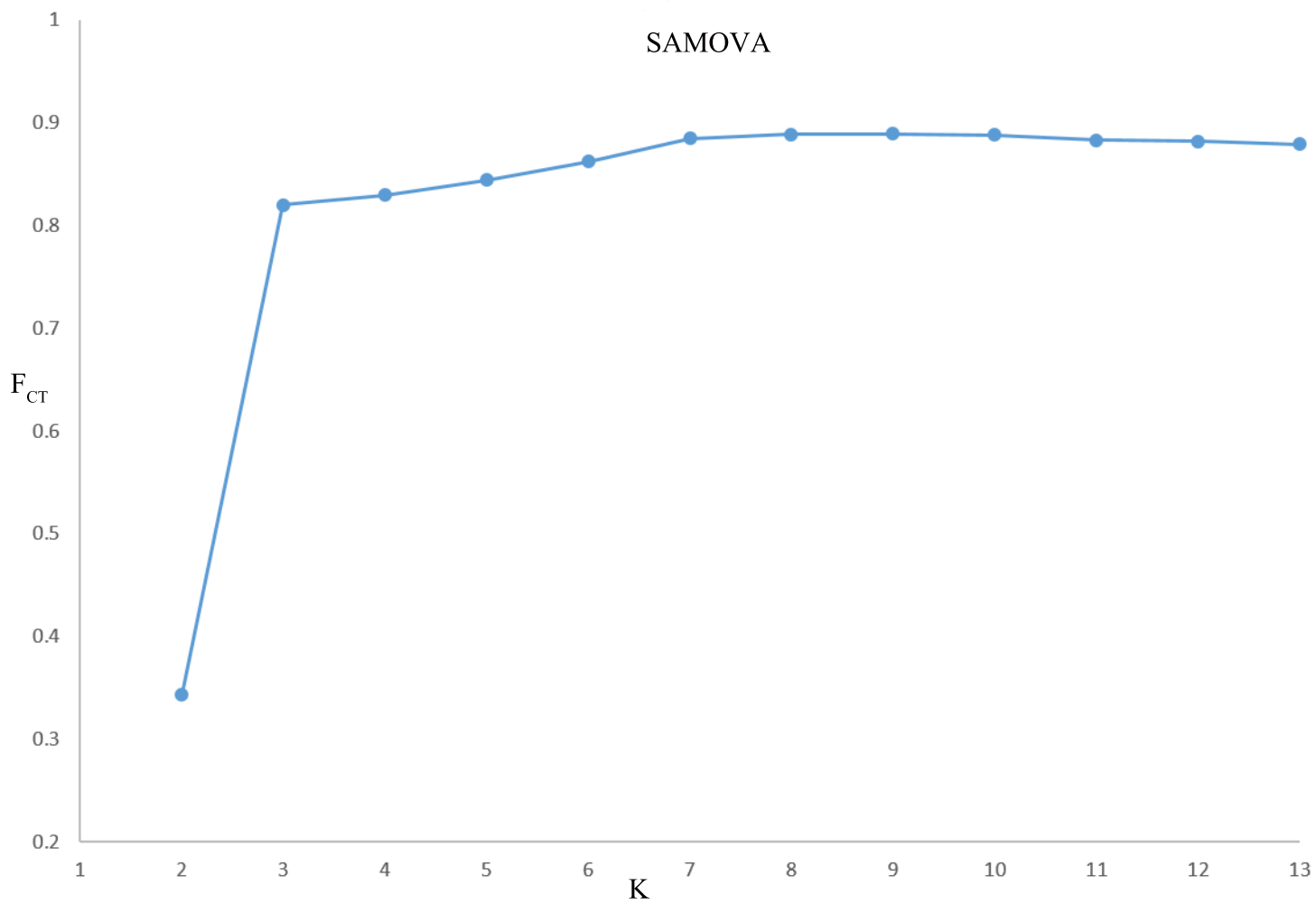

Supplement: Supplementary Figure 4 — FCT distribution of mixed cpDNA sequence data. [file Image4.PDF]

Na [4.31e+03]

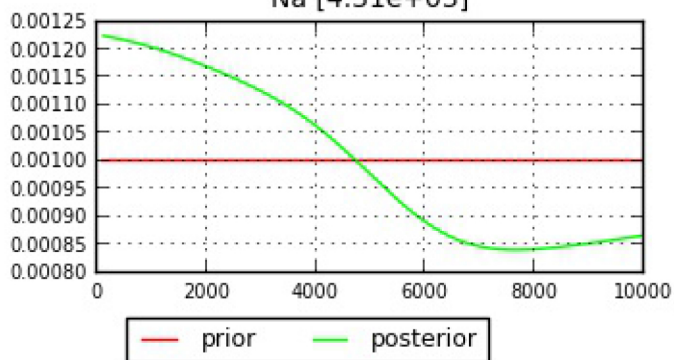

pmic\_1 [3.05e-01]

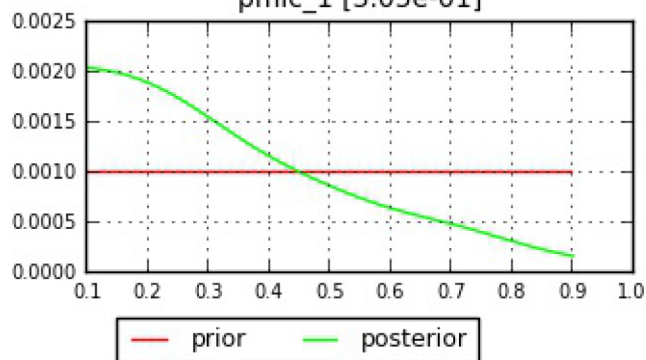

t2 [4.41e+03]

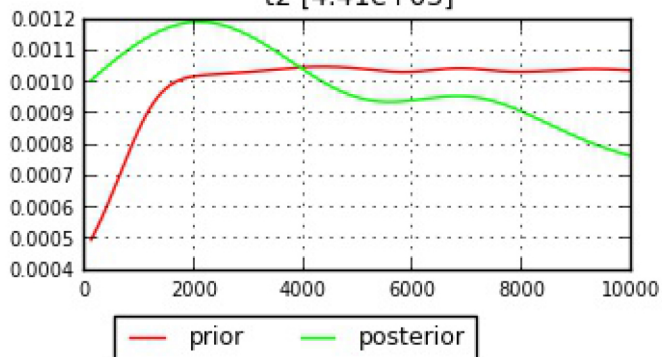

t1 [6.63e+02]

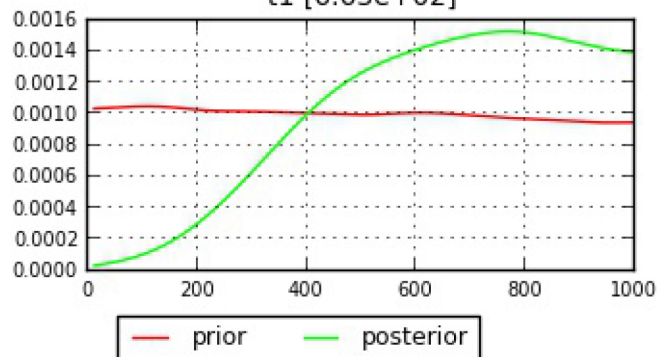 $\mu$ mic\_1 [4.92e-04]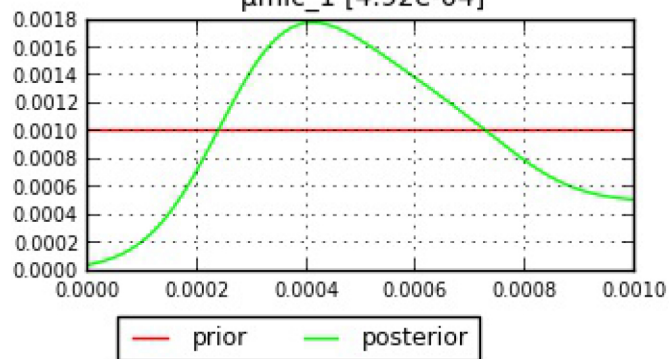

N1 [1.15e+04]

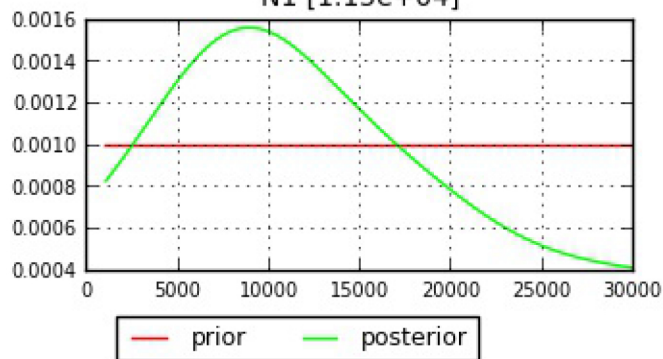

N2 [5.66e+03]

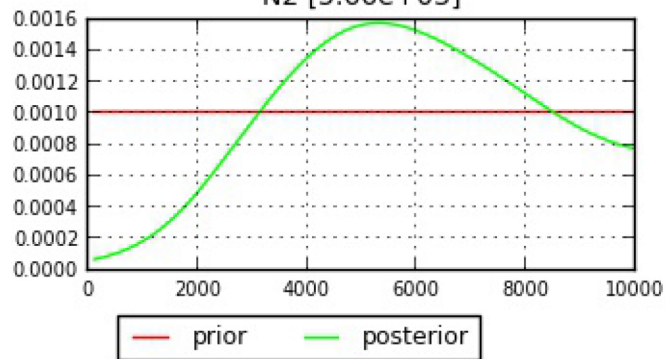

Supplement: Supplementary Figure 6 — Prior and posterior distributions for each parameter in scenario 3, obtained using DIYABC analysis. [file Image6.PDF]
