# Supplementary material for: Phylogeography Study of the Siberian Apricot (Prunus sibirica L.) in Northern China Assessed by Chloroplast Microsatellite and DNA Makers
Source: Front Plant Sci. 2017 Nov 21;8:1989. doi: 10.3389/fpls.2017.01989 (PMC5702509; doi:10.3389/fpls.2017.01989)

K=2 20/20, Mean(LnProb) = -81857.920, Mean(similarity score) = 0.947

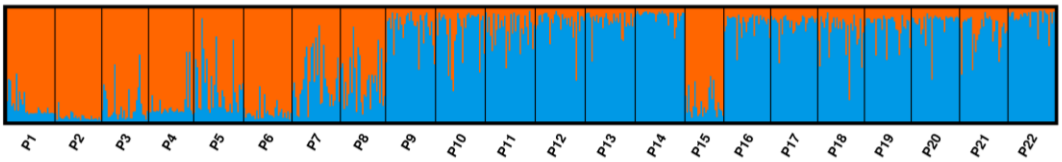

K=3 10/20, Mean(LnProb) = -80849.830, Mean(similarity score) = 0.958

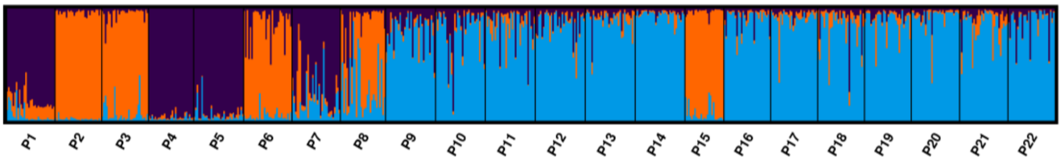

K=3 10/20, Mean(LnProb) = -80940.750, Mean(similarity score) = 0.868

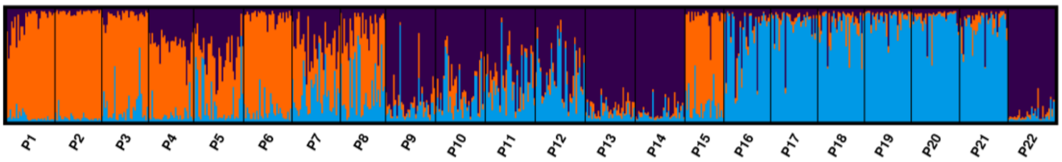

K=4 11/20, Mean(LnProb) = -79935.236, Mean(similarity score) = 0.997

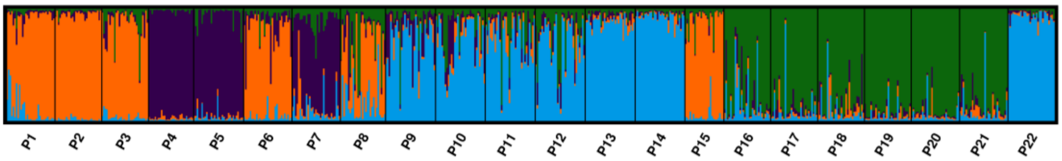

K=4 8/20, Mean(LnProb) = -79921.675, Mean(similarity score) = 0.997

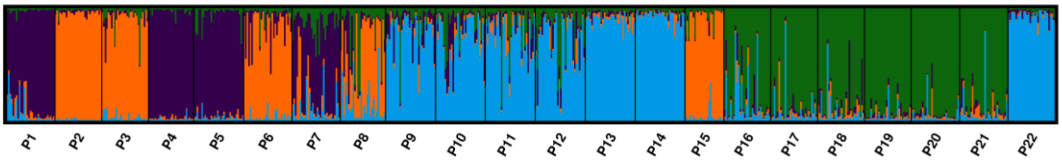

K=5 12/20, Mean(LnProb) = -79292.717, Mean(similarity score) = 0.881

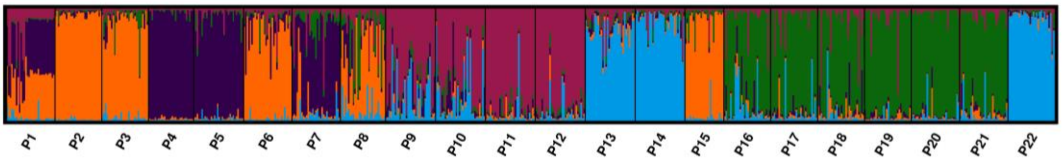

K=5 6/20, Mean(LnProb) = -79220.783, Mean(similarity score) = 0.889

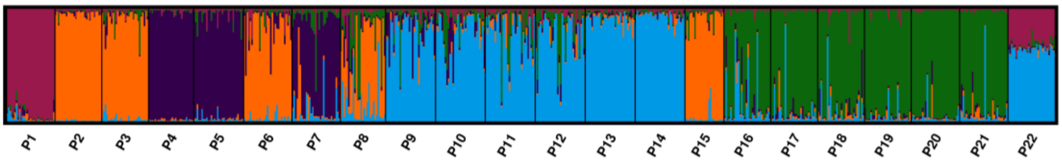

K=5 2/20, Mean(LnProb) = -79397.250, Mean(similarity score) = 0.812

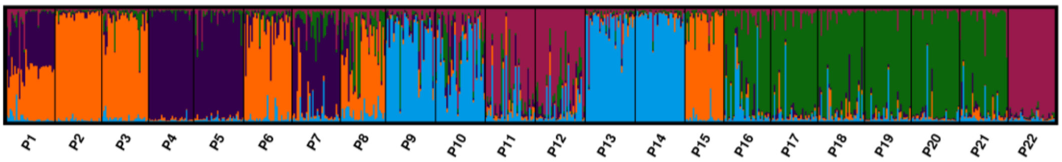

K=6 10/20, Mean(LnProb) = -79612.940, Mean(similarity score) = 0.949

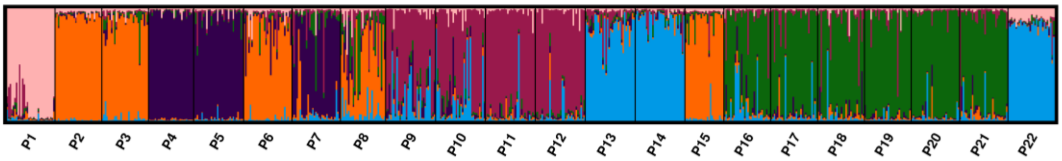

K=6 5/20, Mean(LnProb) = -78826.580, Mean(similarity score) = 0.889

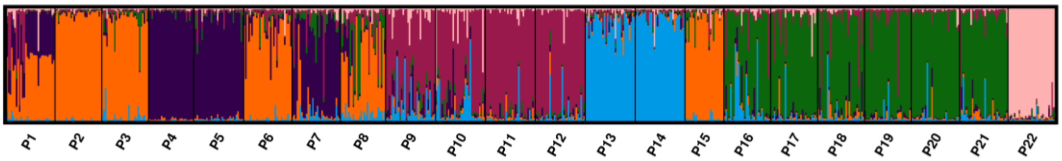

K=6 3/20, Mean(LnProb) = -79081.900, Mean(similarity score) = 0.993

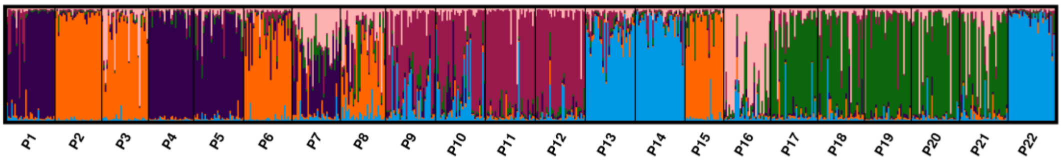

K=6 2/20, Mean(LnProb) = -78980.700, Mean(similarity score) = 0.996

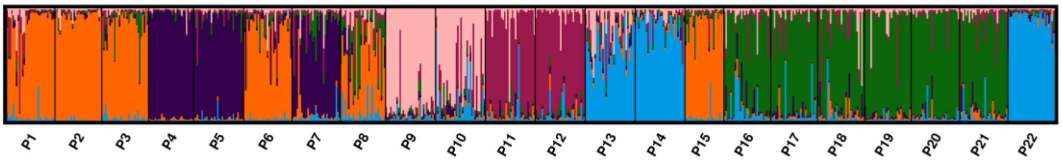

K=7 9/20, Mean(LnProb) = -78328.856, Mean(similarity score) = 0.870

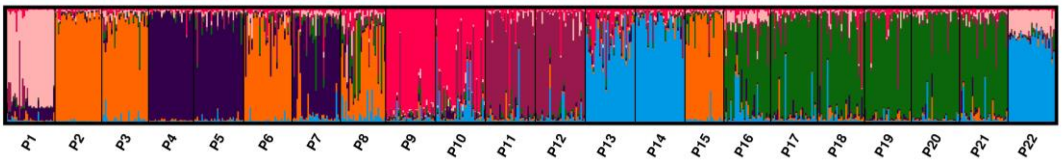

K=7 5/20, Mean(LnProb) = -78103.500, Mean(similarity score) = 0.996

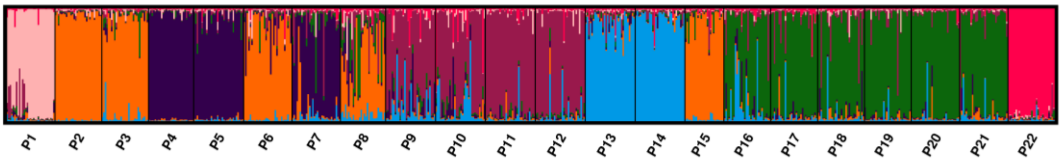

K=7 3/20, Mean(LnProb) = -78313.033, Mean(similarity score) = 0.834

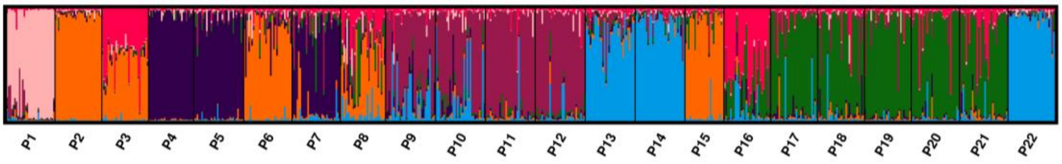

K=7 3/20, Mean(LnProb) = -81342.367, Mean(similarity score) = 0.838

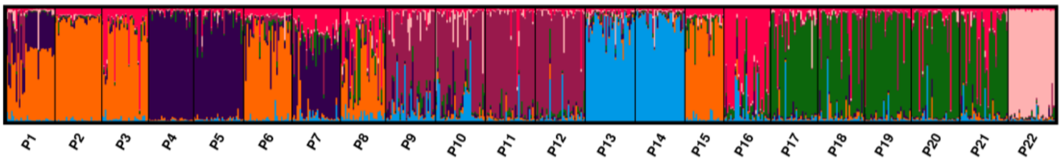

K=8 10/20, Mean(LnProb) = -77883.080, Mean(similarity score) = 0.995

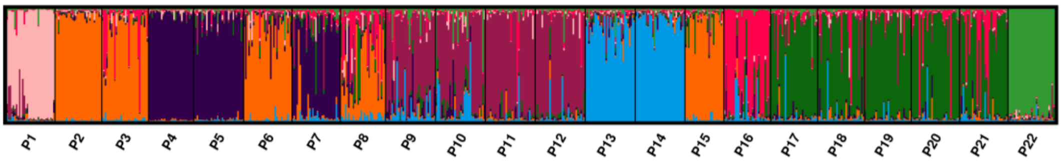

K=8 5/20, Mean(LnProb) = -78008.880, Mean(similarity score) = 0.899

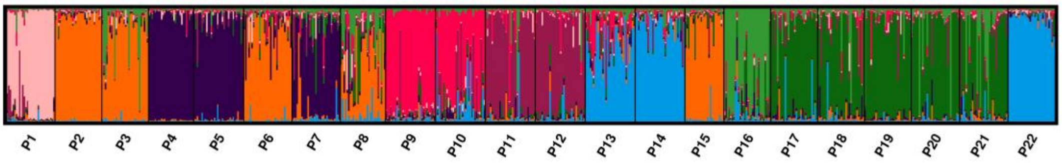

K=8 5/20, Mean(LnProb) = -83828.560, Mean(similarity score) = 0.995

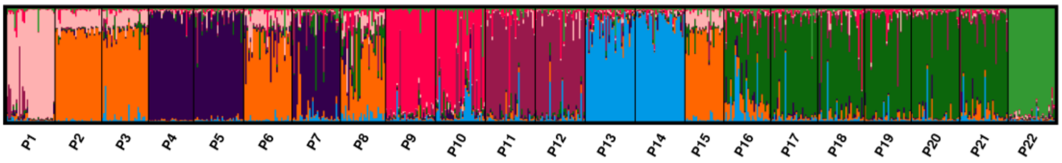

K=9 11/20, Mean(LnProb) = -77800.880, Mean(similarity score) = 0.923

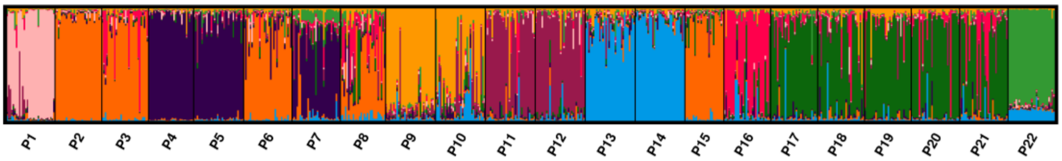

K=9 9/20, Mean(LnProb) = -77822.367, Mean(similarity score) = 0.805

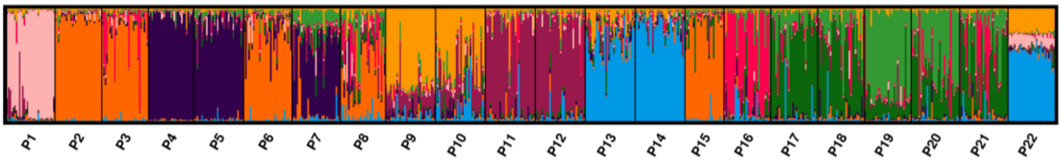

K=10 13/20, Mean(LnProb) = -77372.823, Mean(similarity score) = 0.897

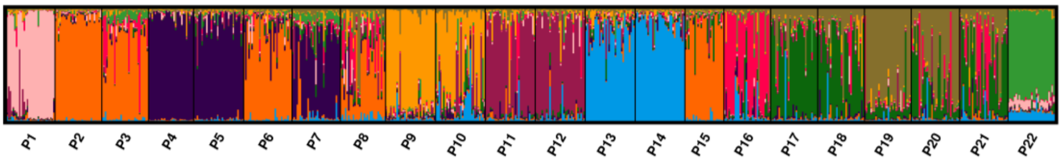

K=10 7/20, Mean(LnProb) = -77154.750, Mean(similarity score) = 0.862

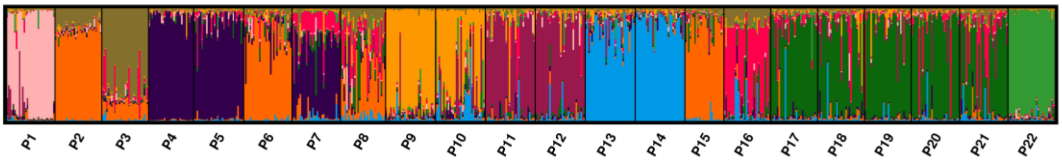

K=11 13/20, Mean(LnProb) = -77386.338, Mean(similarity score) = 0.890

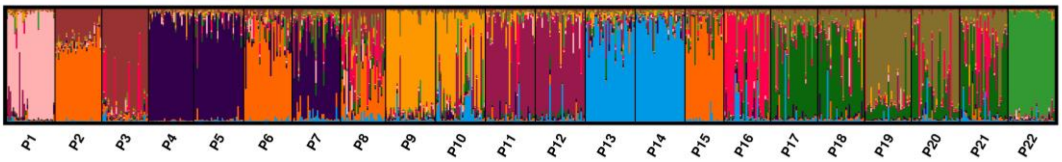

K=11 4/20, Mean(LnProb) = -76881.000, Mean(similarity score) = 0.882

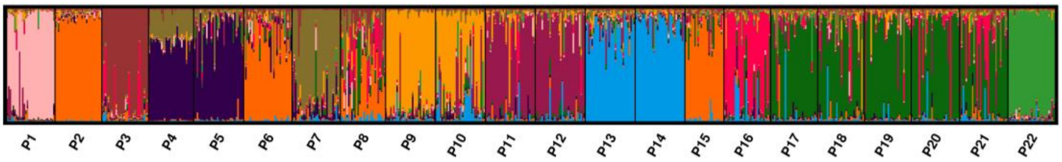

K=11 3/20, Mean(LnProb) = -77126.300, Mean(similarity score) = 0.982

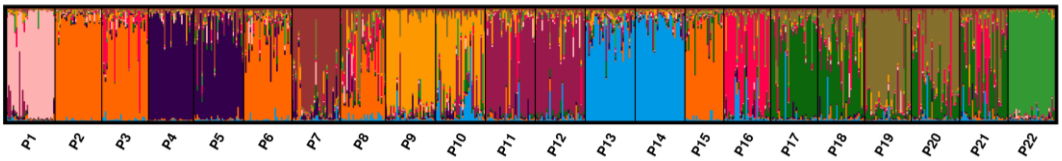

Supplement: Supplementary Figure 5 — The CLUMPAK main pipeline of all clustering. [file Image5.PDF]
