# Supplementary material for: Phylogeography Study of the Siberian Apricot (Prunus sibirica L.) in Northern China Assessed by Chloroplast Microsatellite and DNA Makers
Source: Front Plant Sci. 2017 Nov 21;8:1989. doi: 10.3389/fpls.2017.01989 (PMC5702509; doi:10.3389/fpls.2017.01989)
Supplement: Supplementary file 8 [file Table2.DOCX]

Supplementary Material

Phylogeography of the Siberian apricot (*Prunus sibirica* L.) indicates a climate-related boundary in northern China

**Zhe Wang^1,4†^, Yanfei Zeng^2†^, Zhendong Zhang^1^, Songbai Sheng^1^, Yang Liu^3^, Rongling Wu^4^ and Xiaoming Pang^1*^**

^1^ National Engineering Laboratory for Tree Breeding, Key Laboratory of Genetics and Breeding in Forest Trees and Ornamental Plants, Ministry of Education, Center for Computational Biology, College of Biological Sciences and Biotechnology, Beijing Forestry University, Beijing 100083, China

^2^ State Key Laboratory of Tree Genetics and Breeding, Chinese Academy of Forestry, Beijing 100091, China

^3^ Inner Mongolia Hesheng Ecological Science and Technology Research Institute, Huhhot 011517, China

^4^ Center for Computational Biology, College of Biological Sciences and Biotechnology, Beijing Forestry University, Beijing 100083, China

**^*^Correspondence:**

Xiaoming Pang

Fax: +86-10-62336164

Email: [xmpang@bjfu.edu.cn](mailto:xmpang@bjfu.edu.cn)

^†^These authors contributed equally to this study and share first authorship;

**Supplementary Table2** Clock models compared by AICM.

| Trace | AICM | S.E. | Strict clock | Lognormal relaxed clock | Exponential relaxed clock | Random local clock | Fixed local clock |
| --- | --- | --- | --- | --- | --- | --- | --- |
| Strict clock | 3721.03 | 0.026 | - | 2.1 | 0.43 | 0.766 | 1.082 |
| Lognormal relaxed clock | 3723.13 | 0.025 | -2.1 | - |  |  | -1.019 |
| Exponential relaxed clock | 3721.46 | 0.02 | -0.43 | 1.67 | - |  | 0.651 |
| Random local clock | 3721.796 | 0.03 | -0.766 | 1.334 | -0.336 | - | 0.315 |
| Fixed local clock | 3722.111 | 0.012 | -1.082 | 1.019 | -0.651 | -0.315 | - |

Lower AICM values indicate better model fit. Differences between AICM estimates are reported. Positive values indicate better relative model fit of the row's model compared to the column's model.
