# Supplementary material for: Phylogeography Study of the Siberian Apricot (Prunus sibirica L.) in Northern China Assessed by Chloroplast Microsatellite and DNA Makers
Source: Front Plant Sci. 2017 Nov 21;8:1989. doi: 10.3389/fpls.2017.01989 (PMC5702509; doi:10.3389/fpls.2017.01989)
Supplement: Supplementary file 9 [file Table3.DOCX]

Supplementary Material

Phylogeography of the Siberian apricot (*Prunus sibirica* L.) indicates a climate-related boundary in northern China

**Zhe Wang^1,4†^, Yanfei Zeng^2†^, Zhendong Zhang^1^, Songbai Sheng^1^, Yang Liu^3^, Rongling Wu^4^ and Xiaoming Pang^1*^**

^1^ National Engineering Laboratory for Tree Breeding, Key Laboratory of Genetics and Breeding in Forest Trees and Ornamental Plants, Ministry of Education, Center for Computational Biology, College of Biological Sciences and Biotechnology, Beijing Forestry University, Beijing 100083, China

^2^ State Key Laboratory of Tree Genetics and Breeding, Chinese Academy of Forestry, Beijing 100091, China

^3^ Inner Mongolia Hesheng Ecological Science and Technology Research Institute, Huhhot 011517, China

^4^ Center for Computational Biology, College of Biological Sciences and Biotechnology, Beijing Forestry University, Beijing 100083, China

**^*^Correspondence:**

Xiaoming Pang

Fax: +86-10-62336164

Email: [xmpang@bjfu.edu.cn](mailto:xmpang@bjfu.edu.cn)

^†^These authors contributed equally to this study and share first authorship;

**Supplementary Table3** Haplotype definition and frequency based on the combination of 17 chloroplast microsatellite alleles found in seven polymorphisms.

|  | Freq. | NO. | s.d. | Haplotype: |
| --- | --- | --- | --- | --- |
| S1 | 0.459 | 102 | 0.033523 | 127 225 222 121 153 222 275 |
| S2 | 0.005 | 1 | 0.004505 | 127 226 222 121 153 222 275 |
| S3 | 0.005 | 1 | 0.004505 | 127 225 222 121 153 222 271 |
| S4 | 0.077 | 17 | 0.017888 | 123 226 223 121 153 222 271 |
| S5 | 0.014 | 3 | 0.007767 | 129 225 221 121 153 222 275 |
| S6 | 0.005 | 1 | 0.004505 | 127 225 222 122 153 222 275 |
| S7 | 0.198 | 44 | 0.026816 | 123 226 223 121 153 221 271 |
| S8 | 0.041 | 9 | 0.013267 | 127 225 222 121 154 222 275 |
| S9 | 0.045 | 10 | 0.013951 | 123 226 221 121 153 221 271 |
| S10 | 0.090 | 20 | 0.019259 | 129 225 222 122 153 221 275 |
| S11 | 0.005 | 1 | 0.004505 | 127 225 223 123 153 222 271 |
| S12 | 0.005 | 1 | 0.004505 | 123 226 222 122 153 221 271 |
| S13 | 0.005 | 1 | 0.004505 | 123 225 222 121 153 222 275 |
| S14 | 0.005 | 1 | 0.004505 | 123 226 222 121 153 221 271 |
| S15 | 0.045 | 10 | 0.013951 | 125 226 223 122 153 221 271 |
