# Supplementary material for: Phylogeography Study of the Siberian Apricot (Prunus sibirica L.) in Northern China Assessed by Chloroplast Microsatellite and DNA Makers
Source: Front Plant Sci. 2017 Nov 21;8:1989. doi: 10.3389/fpls.2017.01989 (PMC5702509; doi:10.3389/fpls.2017.01989)
Supplement: Supplementary file 10 [file Table4.DOCX]

Supplementary Material

Phylogeography of the Siberian apricot (*Prunus sibirica* L.) indicates a climate-related boundary in northern China

**Zhe Wang^1,4†^, Yanfei Zeng^2†^, Zhendong Zhang^1^, Songbai Sheng^1^, Yang Liu^3^, Rongling Wu^4^ and Xiaoming Pang^1*^**

^1^ National Engineering Laboratory for Tree Breeding, Key Laboratory of Genetics and Breeding in Forest Trees and Ornamental Plants, Ministry of Education, Center for Computational Biology, College of Biological Sciences and Biotechnology, Beijing Forestry University, Beijing 100083, China

^2^ State Key Laboratory of Tree Genetics and Breeding, Chinese Academy of Forestry, Beijing 100091, China

^3^ Inner Mongolia Hesheng Ecological Science and Technology Research Institute, Huhhot 011517, China

^4^ Center for Computational Biology, College of Biological Sciences and Biotechnology, Beijing Forestry University, Beijing 100083, China

**^*^Correspondence:**

Xiaoming Pang

Fax: +86-10-62336164

Email: [xmpang@bjfu.edu.cn](mailto:xmpang@bjfu.edu.cn)

^†^These authors contributed equally to this study and share first authorship;

**Supplementary Table4** Population genetic diversity revealed by cpSSR markers for 22 Siberian apricot populations.

| **Pop** | **N** | **Ne** | **H** | **He** | **Np** |
| --- | --- | --- | --- | --- | --- |
| P1 | 1 | 1 | 0 | 0 | 0 |
| P2 | 4 | 1.282 | 0.189 | 0.21 | 2 |
| P3 | 2 | 1.031 | 0.026 | 0.029 | 1 |
| P4 | 6 | 1.746 | 0.38 | 0.422 | 3 |
| P5 | 1 | 1 | 0 | 0 | 0 |
| P6 | 2 | 1.132 | 0.069 | 0.076 | 0 |
| P7 | 1 | 1 | 0 | 0 | 0 |
| P8 | 1 | 1 | 0 | 0 | 0 |
| P9 | 1 | 1 | 0 | 0 | 0 |
| P10 | 1 | 1 | 0 | 0 | 0 |
| P11 | 1 | 1 | 0 | 0 | 0 |
| P12 | 1 | 1 | 0 | 0 | 0 |
| P13 | 2 | 1.031 | 0.026 | 0.029 | 1 |
| P14 | 1 | 1 | 0 | 0 | 0 |
| P15 | 1 | 1 | 0 | 0 | 1 |
| P16 | 1 | 1 | 0 | 0 | 0 |
| P17 | 4 | 1.688 | 0.366 | 0.406 | 1 |
| P18 | 1 | 1 | 0 | 0 | 1 |
| P19 | 2 | 1.132 | 0.069 | 0.076 | 0 |
| P20 | 2 | 1.236 | 0.161 | 0.177 | 0 |
| P21 | 1 | 1 | 0 | 0 | 0 |
| P22 | 1 | 1 | 0 | 0 | 0 |
| Average | 1.727 | 1.104 | 0.058 | 0.065 | 0.455 |
